# Combining GRP78 suppression and MK2206-induced Akt inhibition decreases doxorubicin-induced P-glycoprotein expression and mitigates chemoresistance in human osteosarcoma

**DOI:** 10.18632/oncotarget.10890

**Published:** 2016-07-28

**Authors:** Yuan-Zheng Xia, Lei Yang, Gui-Min Xue, Chao Zhang, Chao Guo, Yan-Wei Yang, Shan-Shan Li, Lu-Yong Zhang, Qing-Long Guo, Ling-Yi Kong

**Affiliations:** ^1^ State Key Laboratory of Natural Medicines, Department of Natural Medicinal Chemistry, China Pharmaceutical University, Nanjing 210009, People's Republic of China

**Keywords:** P-gp, GRP78, Akt, multidrug resistance, osteosarcoma

## Abstract

P-glycoprotein (P-gp) overexpression is associated with poor prognosis and drug-resistance in osteosarcoma (OS), but the underlying mechanisms remain incompletely understood. Here, we examined the regulation of P-gp, GRP78, and phospho-Akt in doxorubicin (DOX)-treated OS cells. DOX induced P-gp expression, which was associated with increased GRP78 levels and Akt activation *in vitro* and *in vivo*. Functional analysis showed that Akt induces P-gp and GRP78 expression, which contributes to the DOX-induced Akt activation. Examination of the relationship between Akt and GRP78 demonstrated that GRP78 suppression attenuates the Akt activity in OS parental sensitive and resistant cells, indicating that GRP78 is required for full Akt activity. Inhibition of Akt activity using MK2206 decreased GRP78 expression in OS cells, which enhanced the inhibitory effect of MK2206 on P-gp expression. GRP78 knockdown combined with MK2206 suppressed the development of DOX resistance in OS cells and inhibited the *in vivo* tumor growth in the presence of DOX. These results support the development of novel therapeutic strategies that target GRP78 and Akt to sensitize OS cells for chemotherapy.

## INTRODUCTION

P-glycoprotein (P-gp, also known as ABCB1) overexpression is an important mechanism of inherent and acquired multidrug resistance (MDR) in human osteosarcoma (OS) [1–3]. P-gp levels serve as a prognostic marker and correlate with poor chemotherapeutic response [4–6]. Notably, MDR-associated protein 1 (MRP1) and breast cancer resistance protein (BCRP), which are two eminent ATP-binding cassette (ABC) transporters that modulate anticancer drug uptake and efflux, do not correlate as closely as P-gp with an MDR phenotype [5, 7–9]. P-gp positive variants can be selected from P-gp negative parental uterine sarcoma MES-SA cells after a single exposure to doxorubicin (DOX), demonstrating that chemotherapeutic drugs can lead to the production of MDR cells [10, 11].

Analysis of the *ABCB1* promoter and flanking sequences has revealed that several elements including the heat shock element and Y-box region, also found in the *heat shock protein-70* (*HSP70*) promoter, are located upstream from the transcription initiation site [12, 13]. The glucose-regulated protein 78 (GRP78, also referred to as BiP), a major endoplasmic reticulum (ER) chaperone, is a member of the HSP70 protein family [14]. During ER stress, which usually occurs under cytotoxic chemical stimulation or UV radiation, cells initiate an unfolded protein response (UPR), which employs GRP78 as a necessary regulator to maintain homeostasis of ER function [15, 16]. Overexpression of GRP78 in cancer cells has been associated with drug resistance and enhanced malignancy [17]. P-gp levels positively correlate with GRP78 expression, despite the lack of evidence of direct association between P-gp and GRP78 [5, 18, 19].

The serine/threonine kinase Akt, a primary downstream mediator of phospho-inositide 3-kinase (PI3K) signaling, modulates cell proliferation, migration, survival, and death [20]. Studies have suggested that Akt promotes cell survival by inducing P-gp expression under chemotherapeutic conditions, and that GRP78 promotes Akt-mediated drug resistance after cisplatin administration [16, 21, 22]. MK2206, a small molecule pan-Akt inhibitor, has been evaluated in clinical trials to treat solid tumors, and has been proven safe in humans, and orally effective [16, 20, 23]. Inhibition of Akt activity by MK2206 prevents cisplatin resistance in endometrial cancer cells and reverses drug resistance in human melanoma cells [16, 24].

Multiple strategies to reverse P-gp-mediated MDR have been investigated, including designing inhibitors to block ATP-dependent P-gp activity to block efflux and recover drug accumulation, as well as exploring small molecules to reduce P-gp levels in MDR tumor cells [25–28]. In this study, we investigated the regulation of P-gp, GRP78, and p-Akt in human OS cells and their DOX-resistant sublines, and we examined the generation of DOX chemoresistance *in vitro* and *in vivo*.

## RESULTS

### DOX up-regulates P-gp, GRP78, and p-Akt in human OS cells *in vitro* and *in vivo*

**Figure 1:**
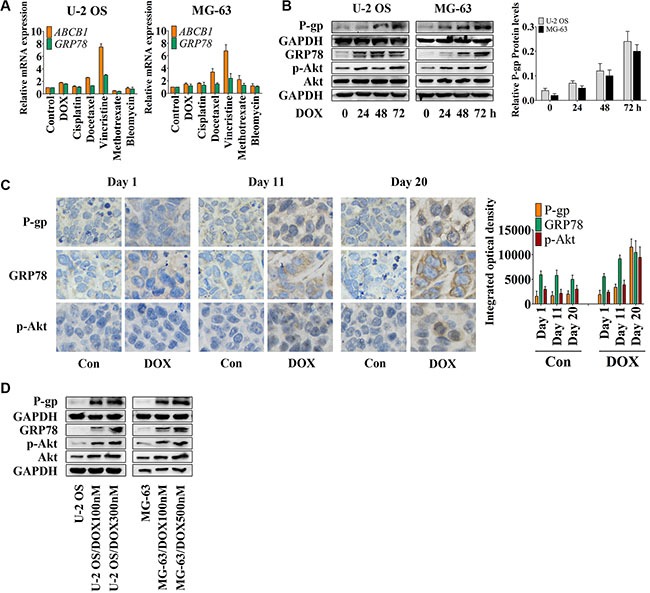
DOX induces P-gp, GRP78, and p-Akt expression *in vitro* and *in vivo* (**A**) U-2 OS and MG-63 cells were treated with conventional chemotherapeutic drugs in the concentration of their IC_50_ values for OS cells for 24 hr and detected by qRT-PCR. The Δ cycle threshold method was used for the calculation of relative differences in mRNA abundance. Data were normalized to the expression of *GAPDH*. The results of real-time RT-PCR were expressed as fold-changes. The normalized value of the target mRNA of the control group is arbitrarily presented as 1 (*n* = 6). (**B**) U-2 OS and MG-63 cells were treated with 1 μM DOX for 0 to 72 hr. The expression of P-gp at the membrane surface and the levels of GRP78, p-Akt and Akt in the whole-cell lysates were detected by Western blot and normalized by GAPDH (*n* = 3). (**C**) Nude mice bearing xenograft tumors were treated with DOX by intraperitoneal injection once every four days. Three mice were selected randomly and sacrificed every day. Xenografts were removed, fixed and paraffin embedded. IHC staining was performed by using P-gp, GRP78 and p-Akt antibodies (×400) (*n* = 6). (**D**) Western blot results of the membrane lysates or total lysates from OS cell lines and their resistant sublines (*n* = 3). Data are represented as mean ± SD.

To assess the effect of conventional chemotherapy on the expression of *ABCB1* and *GRP78*, we treated cells with several chemotherapeutic drugs (as shown in the Figure 1A) for 24 hr, and analyzed the *ABCB1* and *GRP78* expression by quantitative real-time RT-PCR (qRT-PCR). The expression changes of *ABCB1* induced by chemotherapeutic agents were similar to *GRP78* in both U-2 OS and MG-63 cells. DOX, cisplatin, docetaxel, and vincristine, but not methotrexate or bleomycin stimulated *ABCB1* and *GRP78* expression in U-2 OS cells. In MG-63 cells, most of the conventional chemotherapeutic drugs induced the expression of *ABCB1* and *GRP78*.

DOX was selected for further research. Western blot analysis (Figure 1B) showed that DOX induced P-gp expression in a time-dependent manner. With DOX treatment, U-2 OS cells showed increased Akt activity after 24 hr and decreased activity after 48 hr, followed by increased GRP78 levels at 48 hr and decreased levels at 72 hr after treatment. After 48 hr, DOX also significantly induced GRP78 expression in MG-63 cells. Akt activity was notably increased at 24 hr and then showed minimal variation at 48 hr, but was markedly induced at 72 hr subsequently in MG-63 cells. Thus, the observed increase in Akt activity preceded the induction of GRP78 expression after 24 hr.

In an MG-63 xenograft mouse model, immunohistochemistry (IHC) showed up-regulation of P-gp, GRP78, and p-Akt at day 11 after the treatment; a further increase was observed at the end of the treatment (Figure 1C). To confirm these data *in vitro*, U-2 OS and MG-63 DOX-resistant sublines were established [2, 29]. P-gp, GRP78, p-Akt, and Akt protein levels correlated with the concentration of DOX, which was added to the culture medium to maintain the resistance of the established sublines (Figure 1D). The profiles of OS resistant cells and OS parental sensitive cells, including the IC_50_ values, the growth curves and the DOX accumulation assay, are shown in Table 1 and Figure S1A and S1B. Previous studies also demonstrated the resistant profiles of MG-63 resistant subline [2, 29]. In addition, the OS resistant sublines showed slight resistance to MK2206 compared with their parental cell lines (Table 1).

**Table 1:** IC_50_ ± SD (μM) profile for each chemical in OS cell lines

	Cell line			
Chemical	U-2 OS	U-2 OS/DOX	MG-63	MG-63/DOX
Bleomycin	6.53 ± 1.14	35.82 ± 1.63	4.57 ± 0.59	30.81 ± 1.12
Cisplatin	3.58 ± 1.09	10.72 ± 1.68	2.13 ± 0.75	11.32 ± 1.81
Docetaxel	0.73 ± 0.49	4.58 ± 1.37	1.35 ± 0.82	6.37 ± 1.03
**DOX**	**0.92 ± 0.51**	**38.26 ± 1.85**	**1.05 ± 0.31**	**44.96 ± 1.38**
Methotrexate	0.71 ± 0.13	4.76 ± 0.52	1.17 ± 0.42	5.07 ± 0.93
**MK2206**	**8.75** ± **1.68**	**15.63** ± **2.09**	**9.59** ± **1.73**	**17.35** ± **1.52**
Vincristine	1.57 ± 0.61	8.96 ± 1.64	0.62 ± 0.15	7.97 ± 1.24

### *GRP78* silencing inhibits DOX-induced P-gp expression in OS cells and their DOX-resistant sublines

**Figure 2:**
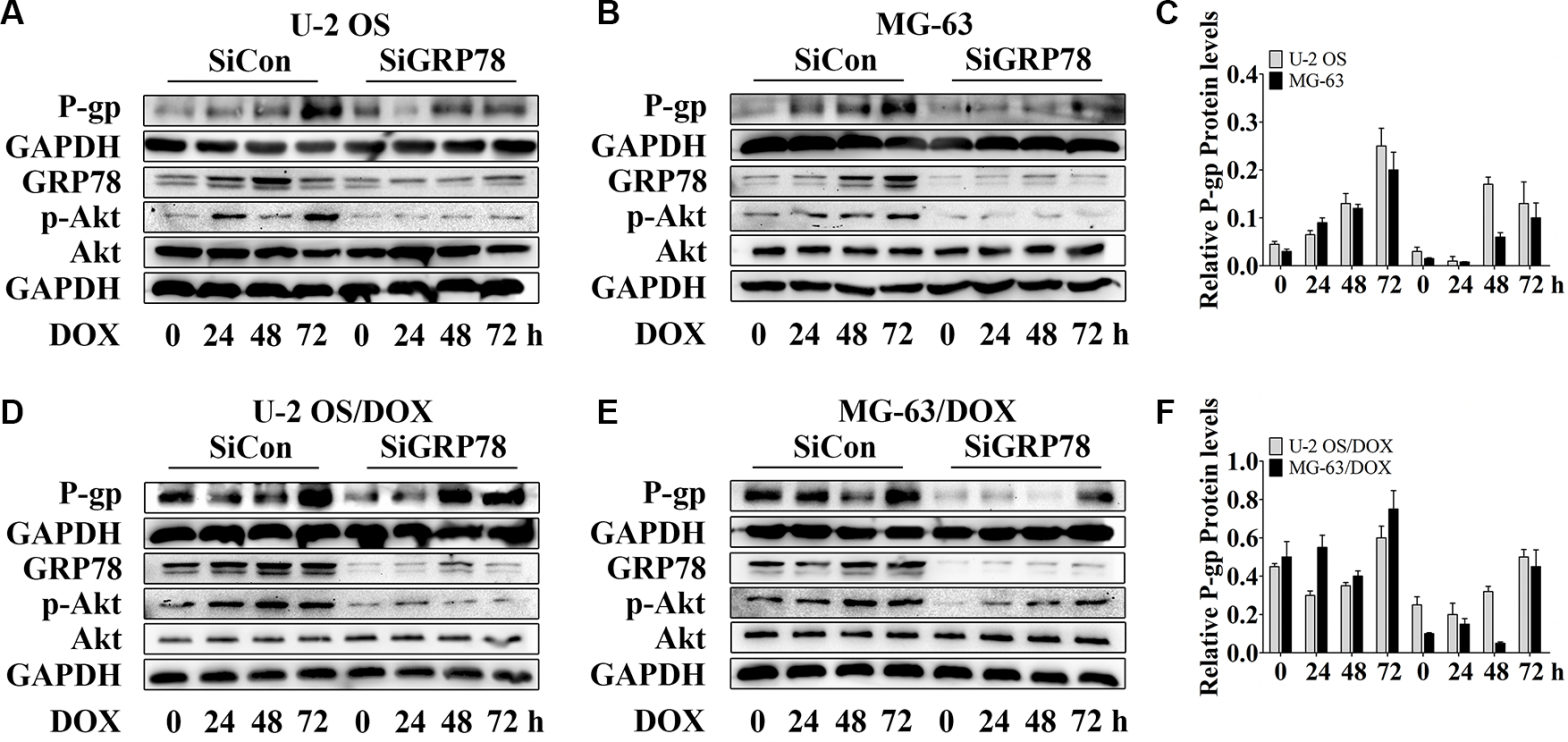
Knockdown of GRP78 slightly prevents DOX-induced P-gp expression in OS parental cell lines and resistant sublines (**A**–**F**) OS cells and their resistant sublines were transfected transiently with siGRP78 or control siRNA for 24 hr and then treated with 1 μM DOX for 0 to 72 hr. Western blot analysis was performed with GAPDH as loading control. Data are represented as mean ± SD (*n* = 3).

Interestingly, the levels of P-gp and GRP78 were up-regulated after the DOX treatment. However, P-gp does not directly associate with GRP78 [30]. To determine whether GRP78 enhances the levels of P-gp in response to DOX treatment, GRP78 knockdown was performed by siRNA, and changes in P-gp expression were determined. Significant increase of P-gp and p-Akt expression in OS parental sensitive cell lines was observed with the induction of 0.5 μM DOX while the resistant sublines responded to 1 μM DOX (data not shown). Thus, 1 μM DOX was used in the entire study. After knockdown, GRP78 expression was suppressed efficiently. P-gp levels were reduced moderately in parental cells (Figure 2A–2C, lanes 1 and 5) and significantly in DOX-resistant cells (Figure 2D–2F, lanes 1 and 5), in comparison with siRNA controls under normal growth conditions. DOX treatment induced a continuous increase in P-gp from 0 to 72 hr in parental sensitive cells (Figure 2A–2C, lanes 1 to 4), but not in DOX-resistant cells (Figure 2D–2F, lanes 1 to 4). P-gp levels were significantly induced at 72 hr in DOX-resistant cells in control (Figure 2D–2F, lane 4). Comparison of the effects of the knockdown on each cell line showed that the loss of GRP78 mildly inhibited P-gp expression during DOX treatment (Figure 2A–2F, lanes 5 to 8).

Moreover, GRP78 knockdown resulted in a slight decrease in constitutive Akt activity in parental and DOX-resistant cells (Figure 2A–2F, lanes 1 and 5). After incubation, DOX stimulated Akt phosphorylation within 24 hr, followed by increasing GRP78 levels at 48 hr in MG-63/DOX cells in control, but not in U-2 OS/DOX cells (Figure 2D–2F, lanes 1 to 4). During DOX treatment of knockdown cells, Akt activity increased slightly or rarely (Figure 2A–2F, lanes 5 to 8). These results indicate that GRP78 contributes to Akt phosphorylation in DOX-treated OS cells.

### MK2206 inhibits DOX-induced P-gp expression in parental sensitive cells and resistant sublines

**Figure 3:**
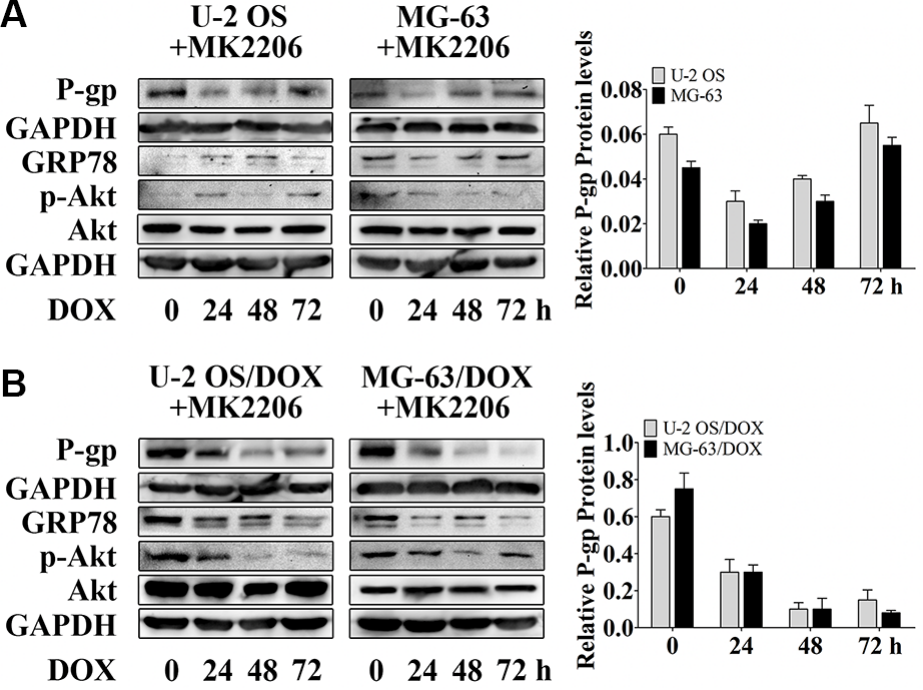
MK2206 inhibits DOX-induced P-gp expression in OS parental cell lines and resistant sublines (**A** and **B**) OS cells and their resistant sublines were treated with 30 nM MK2206 for 4 hr followed by treating with 1 μM DOX for 0 to 72 hr. Western blot analysis was performed with GAPDH as loading control. Data are represented as mean ± SD (*n* = 3).

To confirm the alteration of P-gp expression associated with Akt activity, we used MK2206 to block Akt activation. MK2206 at 50 nM and higher concentrations, inhibits Akt phosphorylation (data not shown), and may cover up the contribution of other factors to Akt activity. In order to study the function of GRP78 in Akt phosphorylation, 30 nM MK2206 was used. As expected, MK2206 inhibited the DOX-induced P-gp up-regulation in parental sensitive cells (Figure 3A, lanes 1 and 4), and particularly in DOX-resistant cells (Figure 3B, lanes 1 and 4). This inhibition was more pronounced than in GRP78 knockdown cells (Figure 2A–2F, lanes 5 and 8). In the presence of MK2206, DOX barely increased the P-gp levels in parental cells (Figure 3A) and in DOX-resistant cells (Figure 3B).

In addition, Akt inhibition by MK2206 decreased the GRP78 levels in parental and DOX-resistant OS cells (Figure 3A and 3B, lanes 1 to 4). The expression of GRP78 in response to DOX was negative in U-2 OS, U-2 OS/DOX and MG-63/DOX cells, but not in MG-63 cells (Figure 3A and 3B, lanes 2 to 4). These data indicate that the DOX-induced GRP78 expression is regulated by Akt in OS cells.

### GRP78 suppression potentiates the inhibitory effect of MK2206 on P-gp expression

**Figure 4:**
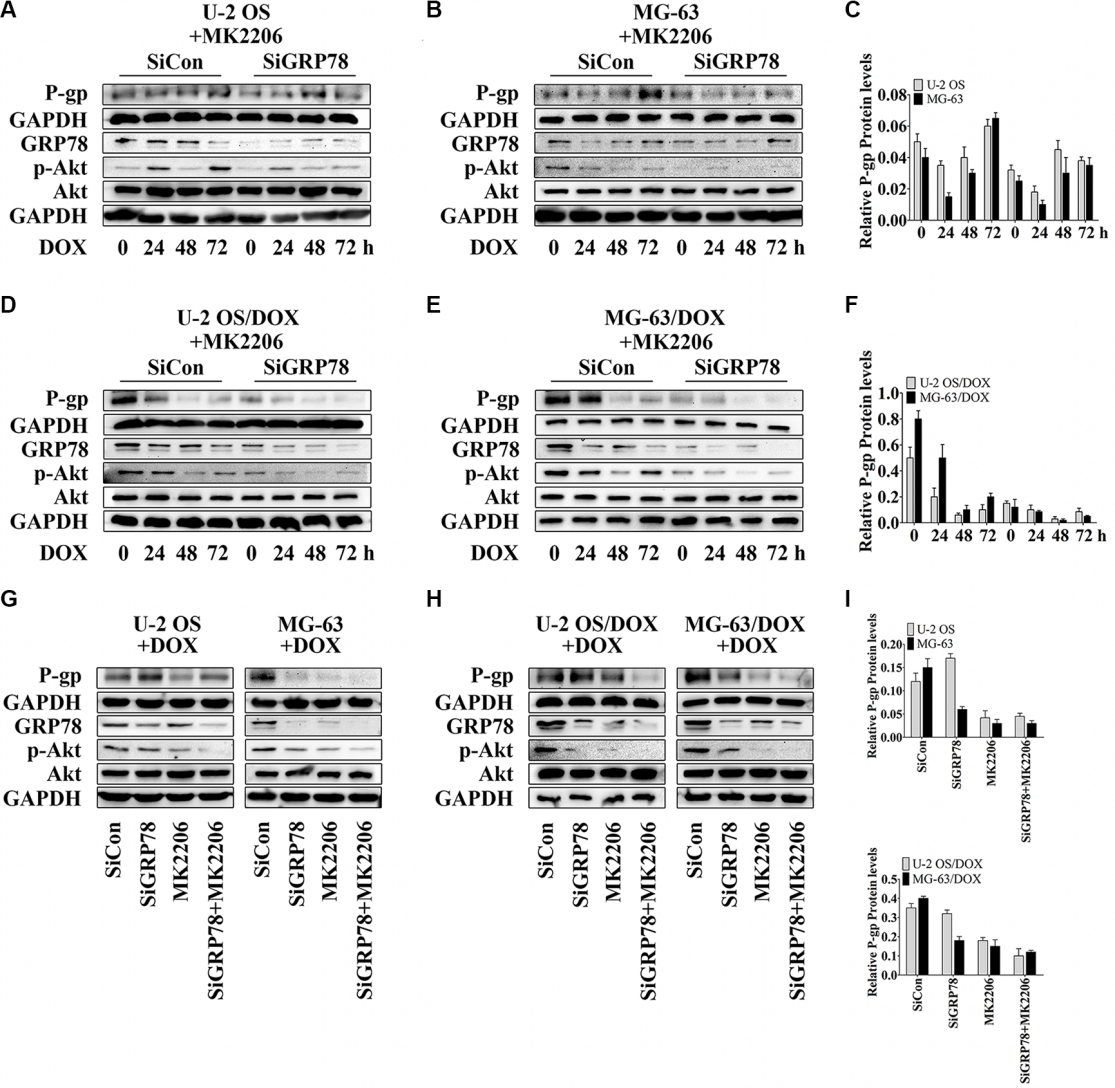
GRP78 inhibition enhances the inhibitory effect of MK2206 on P-gp expression (**A**–**F**) Cells were transfected transiently with siGRP78 or control siRNA for 24 hr. Subsequently, cells were incubated with 30 nM MK2206 for 4 hr followed by treating with 1 μM DOX for 0 to 72 hr. (**G**–**I**) Cells were transfected transiently with siGRP78 or control siRNA for 24 hr, and then incubated 4 hours with or without 30 nM MK2206, followed by treatment with 1 μM DOX for 48 hr. Protein levels in the membrane lysates or total lysates of OS cell lines and their resistant sublines were detected by Western blot using GAPDH as loading control. Data are represented as mean ± SD (*n* = 3).

We suppressed GRP78 prior to MK2206 incubation, as GRP78 may be phosphorylated by Akt. GRP78 inhibition further potentiated the suppressive effect of MK2206 on P-gp levels induced by DOX (Figure 4A–4F, lanes 5 to 8) in comparison with MK2206 treatment alone (Figure 4A–4F, lanes 1 to 4), particularly in DOX-resistant OS cells (Figure 4D–4F, lanes 5 to 8).

Furthermore, Akt inhibition by MK2206 after knockdown of GRP78 appeared to be the most effective in reducing GRP78 levels and Akt phosphorylation (Figure 4A–4F, lanes 1 and 5) and suppressed the DOX-induced GRP78 expression and phospho-Akt levels (Figure 4A–4F, lanes 5 to 8).

To investigate P-gp levels in three treatment conditions, we selected the time point of 48 hr. At this point, we not only tested the cytotoxicity of DOX but also found that with combined MK2206 and GRP78 inhibition, DOX showed the most powerful cytotoxicity in parental OS cells, particularly in DOX-resistant cells (data not shown). Western blotting showed that the suppression of Akt activity and GRP78 levels reduced P-gp expression after DOX treatment for 48 hr in DOX-resistant OS cells (Figure 4H and 4I, lanes 3 and 4). In parental sensitive OS cells, P-gp expression was not changed between MK2206 alone and the combined treatment of MK2206 and GRP78 inhibition (Figure 4G and 4I, lanes 3 and 4). As expected, knockdown of GRP78 had a weak effect on reducing P-gp levels after DOX stimulation at 48 hr (Figure 4G–4I, lanes 3 and 4).

GRP78 suppression combined with MK2206 inhibited GRP78 levels and Akt activity more potently than GRP78 suppression (Figure 4G and 4H, lanes 2 and 4) or MK2206 treatment (Figure 4G and 4H, lanes 3 and 4) alone. In addition, MK2206 not only inhibited Akt activity, but also suppressed the DOX-induced GRP78 expression (Figure 4G and 4H, lane3). These findings indicate that GRP78 suppression inhibits the DOX-induced P-gp expression, and increases the MK2206 inhibitory effect on the DOX-induced P-gp levels.

### Combining GRP78 suppression with MK2206 alleviates DOX-induced drug resistance *in vitro* and sensitizes OS cells to DOX in xenograft mouse model

**Figure 5:**
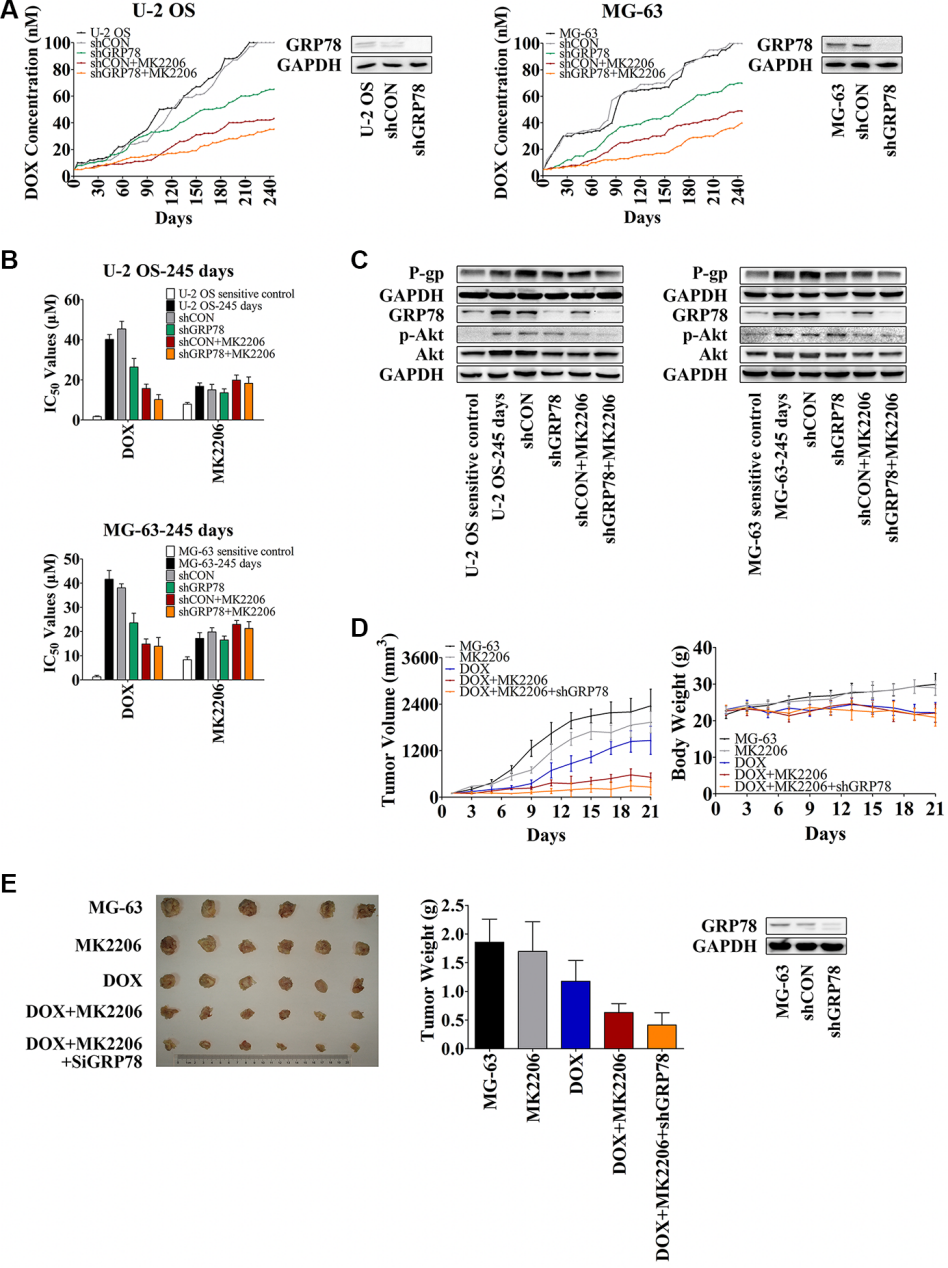
Knockdown of GRP78 combined with MK2206 inhibits the development of DOX-induced chemoresistance in OS cells *in vitro* and *in vivo* (**A**) U-2 OS or MG-63 cells were seeded into 12-well culture plates at 0.5 × 10^5^ cells per well overnight. Subsequently, the cells were pretreated with or without 1 mL of complete media containing 5 μg/mL Polybrene for 5-min. The cells were treated with or without shRNA lentiviral particles for GRP78 or control for 24 hr. Then fresh media without Polybrene was placed and cells were treated with 5 nM DOX and/or 30 nM MK2206. When growth of the cells reached 90% confluence, the cells were harvested and reseeded at the density of 0.5 × 10^5^ cells/well, and the DOX concentration increased. Three parallel experiments in each group were performed simultaneously. The experiments were carried out for 245 days (*n* = 3). (**B**) The IC_50_ values of DOX and MK2206 for each cell line shown in Figure 5A. Cells were seeded into 96-well plates at the density of 3500 cells/well. The next day, concentration gradient of chemicals was added and cells were incubated for further 48 hr. MTT assay was performed (*n* = 3). (**C**) Protein levels in the membrane lysates or total lysates of OS cells were detected by Western blot with GAPDH as loading control. OS parental sensitive cell lines were used as negative control (*n* = 3). (**D** and **E**) MG-63 cells were infected with the lentiviral constructs. After replacing the fresh medium, cells were diluted to 1:3 for selecting stable clones expressing the shRNA by 5 μg/mL puromycin dihydrochloride. Four weeks later, resistant colonies were picked and expanded for xenograft experiments. Three million MG-63 cells were stably transfected with shGRP78, or control lentiviral particles were suspended in 1:2 PBS/Matrigel in a total volume of 200 μL and subcutaneously injected into the right forelimb of each mouse. When mean tumor volume reached approximately 100 mm^3^, 30 mice were randomized into five groups (*n* = 6/group) with approximately equivalent ranges of tumor volume between groups. Mice were treated with MK2206 (60 mg/kg) formulated in 30% Captisol by oral gavage thrice a week. DOX (hydrochloride) (2 mg/kg) was administered by intraperitoneal injection once every 4 days. The control group was treated with 30% Captisol by oral gavage thrice a week. Tumor growth was measured with calipers every other day, and tumor volume was calculated using the following formula: volume (mm^3^) = length × width^2^ × 0.5. After 21 days, the animals were sacrificed, and xenografts were removed and weighed (*n* = 6). Data are represented as mean ± SD.

To understand the relationship between GRP78 expression and Akt activity in mediating resistance, OS cells were treated with GRP78 shRNA and/or MK2206 and subjected to increasing DOX concentrations. After 8 months of DOX alone, U-2 OS and MG-63 cells with or without shRNA control treatment grew steadily in the presence of 100 nM DOX in a culture medium (Figure 5A, black and gray curves). In contrast, OS cells cultured with DOX combined with shGRP78 or MK2206 plus shRNA control showed decreased resistance and were unable to tolerate >100 nM DOX in the culture medium. Specifically, the IC_50_ values were 65 and 42 nM DOX for shGRP78, and MK2206 plus shCON in U-2 OS cells, respectively, and 72 and 49 nM DOX for the same conditions as above in MG-63 cells (Figure 5A, green and brown curves). Notably, under shGRP78 plus MK2206 treatment, cells were more sensitive and did not proliferate in the medium containing > 35 nM DOX (U-2 OS cells) or > 40 nM DOX (MG-63 cells) (Figure 5A, orange curves). The IC_50_ values of DOX correlated with the results in Figure 5A. However, the cells treated with MK2206 were more resistant to MK2206 after 245 days incubation (Figure 5B). Levels of P-gp, GRP78, p-Akt, and Akt were increased after 245 days of DOX treatment (Figure 5C). Not surprisingly, P-gp expression was not increased after treatment with shGRP78 combined with MK2206.

To investigate the role of GRP78 and Akt in developing DOX resistance *in vivo*, we used an MG-63 xenograft mouse model. As described in the Methods, mice were treated for 21 days. As expected, mice treated with DOX had reduced tumor weight and tumor volume compared to control untreated mice (Figure 5D, blue curve in the left panel, and Figure 5E, blue column) or compared to mice treated with MK2206 alone (Figure 5D, gray curve in the left panel, and Figure 5E, gray column). Significant body weight reduction was observed after DOX treatment (Figure 5D, blue curve in the right panel) but not MK2206 (Figure 5D, gray curve in the right panel). MK2206 combined with shGRP78 enhanced the sensitivity of xenograft to DOX. Mice treated with the triple combination had the smallest tumor growth than the other treatment groups (Figure 5D and 5E). Importantly, neither MK2206 nor MK2206 combined with shGRP78 decreased the body weight, indicating that toxicity originated mainly from the DOX administration (Figure 5D, right panel). After 21 days, one mouse died after DOX single injection. Together, these results indicate that the emergence of DOX resistance is regulated partly by the activated Akt and overexpressed GRP78. Combined inhibition of Akt phosphorylation and GRP78 expression does not only increase the DOX sensitivity of OS cells, but also slows down the occurrence of resistance.

## DISCUSSION

DOX, a topoisomerase II inhibitor that is widely used in clinical treatments, is a substrate of P-gp in drug-resistant OS cells [2, 29, 31]. Appropriate stimulation by DOX increases Akt activity and activation of the UPR process, such as the up-regulated GRP78 levels in our study (Figure 1B and 1D). This activation facilitates and maintains high levels of P-gp, particularly in the DOX-resistant sublines (Figure 2D and 2E), and assists the escape of OS cells from DOX-based chemotherapy.

Akt can be activated by phosphorylation at Ser473, Thr308, or both [21, 32, 33]. However, to respond to anticancer drugs, including topoisomerase I or II inhibitors and microtubule stabilizers, Akt is phosphorylated at Ser473 but not at Thr308 [21, 33]. Thus, after DOX treatment, Akt is activated by phosphorylation at Ser473 *in vitro* and *in vivo*. The increased levels of phospho-Akt parallel the enhanced resistance to DOX in OS cells. ABC drug transporters are regulated by Akt-mediated signaling [21]. In other cancers, activation of Akt signaling can promote the development of drug-resistance through up-regulation of Akt-dependent P-gp expression [21, 34]. In addition, Akt is critical for inducing and maintaining GRP78 expression. Suppressing Akt activity prevents drug-induced GRP78 response [16]. Hence, after Akt activity is suppressed by a small-molecule pan-Akt inhibitor, MK2206, both P-gp and GRP78 are blocked (Figures 3 and 4, lanes 1 and 2). These findings suggest that P-gp and GRP78 are downstream targets in the Akt signaling, and that Akt acts as one of the major regulators of P-gp and GRP78 expression. Furthermore, our study not only demonstrates that Akt induces GRP78, but also indicates that GRP78 activates Akt, which requires GRP78 for DOX response (Figure 2).

In addition, our analysis of the relationship between P-gp and GRP78 shows that GRP78 induces P-gp expression under DOX-stimulated condition. We hypothesize that Akt mediates the P-gp induction, and that GRP78 enhances the regulatory effect of Akt on P-gp expression. If our assumption is true, the response of P-gp levels to DOX may be negative or the weakest after knockdown of GRP78 and treatment with MK2206. Our findings suggest that P-gp levels show minimal increase, particularly in DOX-resistant OS cells, during DOX incubation in the absence of GRP78 and phospho-Akt (Figure 4). Thus, under DOX stimulation, GRP78 promotes Akt phosphorylation and enhances Akt-mediated P-gp expression. Given that OS resistant cells express higher levels of P-gp than OS parental cells, we assume that the basal levels of P-gp can be easily detected.

At present, studies of P-gp inhibitors focus on three mechanisms: (i) inhibiting the drug binding site either competitively, noncompetitively, or allosterically; (ii) inhibiting ATP hydrolysis, which is required for P-gp function; and (iii) influencing integrity of cell membrane lipids [35, 36]. However, the factors determining the selectivity of P-gp binding site have not been identified. In addition, safety evaluation of P-gp inhibitors combined with chemotherapeutic drugs would be undesired and not possible to prediction [36–39]. Our analysis demonstrates that the knockdown of GRP78 combined with MK2206 inhibits initial and DOX-induced P-gp levels, indicating the indirectly reverse effect on drug resistance in OS cells (Figure 4).

All three isoforms of Akt: Akt1, Akt2, and Akt3 show clear and distinguishable functions and locations [40]. Current clinical trials on Akt inhibitors target all three Akt isoforms. MK2206, an allosteric Akt inhibitor, is a leading clinical candidate, which has shown only moderate efficacy both alone and in combination with other chemicals to date [41]. The developmental curves of resistant OS cell lines indicate that OS cells treated with shGRP78, MK2206, or both show resistance to DOX by day 245 compared to day 0.

In summary, our study demonstrates that Akt and GRP78 are two important regulators of DOX-induced P-gp expression. We show for the first time that the inhibition of Akt and GRP78 decreases the DOX-induced P-gp expression, and alleviates the chemoresistance of OS cells. Furthermore, our results indicate that GRP78 and Akt mediate the sensitivity of OS cells to DOX, probably by inhibiting the P-gp expression. Suppression of GRP78 offers a potential target and promotes the development of specific antibody therapies targeting the GRP78 expression in tumors, which can be treated with chemotherapeutic drugs and Akt inhibitors.

## MATERIALS AND METHODS

### Cell culture and reagents

Human OS cell lines U-2 OS and MG-63 were purchased from the Typical Culture Preservation Commission Cell Bank (Shanghai, China). MDR variants U-2 OS/DOX and MG-63/DOX were established by exposure to DOX (Santa Cruz Biotechnology, Santa Cruz, CA, USA) at a concentration of 5 nM (0.5% of the concentration required to inhibit growth by 50%), as described previously [2]. U-2 OS cell line and its resistant subline were cultured in RPMI-1640 (Hyclone, Beijing, China) supplemented with 10% fetal bovine serum (FBS, Life Technologies, Grand Island, NY, USA). MG-63 cell line and its resistant variant were maintained in MEM (Life Technologies) in the presence of 10% heat-inactivated FBS. All cell lines were incubated at 37°C in a humidified atmosphere containing 5% CO_2_. U-2 OS/DOX cells cultured in culture medium containing 100 or 300 nM DOX were represented as U-2 OS/DOX 100 nM or U-2 OS/DOX 300 nM. MG-63/DOX cells maintained in the medium involving 100 or 500 nM DOX were denoted by MG-63/DOX 100 nM or MG-63/DOX 500 nM. In this article, unless otherwise stated, U-2 OS/DOX 100 nM and MG-63/DOX 100 nM are denoted by U-2 OS/DOX and MG-63/DOX respectively. Cisplatin and docetaxel were purchased from Sigma-Aldrich (St. Louis, MO, USA). Vincristine, methotrexate, and bleomycin were obtained from Santa Cruz Biotechnology. MK-2206 was purchased from BioVision Incorporated (Milpitas, CA, USA). DOX (hydrochloride) was obtained from Cayman Chemical Company (Ann Arbor, MI, USA). GRP78 shRNA lentiviral particles (sc-44261-V, Santa Cruz Biotechnology) is a pool of concentrated, transduction-ready viral particles containing 3 target-specific constructs that encode 19–25 nt shRNA designed to knock down gene expression; control shRNA (sc-108080, Santa Cruz Biotechnology) is recommended by Santa Cruz Biotechnology as a negative control for GRP78 knockdown.

### qRT-PCR

qRT-PCR was performed as previously described [2, 29]. The sequences of primers used were as follows: *ABCB1*, 5′-AGAGTCAAGGAGCATGGCAC-3′ (sense) and 5′-ACAGTCAGAGTTCACTGGCG-3′ (antisense); *GRP78*, 5′-GAACGTCTGATTGGCGATGC-3′ (sense) and 5′-ACCACCTTGAACGGCAAGAA-3′ (antisense); and *GAPDH*, 5′-GAAAGCCTGCCGGTGACTAA-3′ (sense) and 5′-AGGAAAAGCATCACCCGGAG-3′ (antisense).

### Western blot analysis

Protein levels were analyzed by Western blotting as described previously [2]. Specifically, 18 μg of membrane protein samples and 38 μg of total protein samples were extracted and separated by 10% sodium dodecyl sulfate–polyacrylamide gel electrophoresis. The primary antibodies for P-gp, GRP78, Akt and phosphorylated (Ser473)-Akt (p-Akt) were purchased from Cell Signaling Technology (Danvers, MA, USA) and used at 1:1000 dilution.

### Transient and lentiviral shRNA knockdowns

For Western blot detection, cells were transfected transiently with siRNA as previously described [2, 16]. The sequence of siGRP78 is 5′-ggagcgcauugauacuagatt-3′ and siCon is 5′-aaggagacguauagcaacggu-3′. Transfection was performed using the Lipofectamine 2000 Reagent (Life Technologies) in U-2 OS and U-2 OS/DOX cells and the Super Electroporator NEPA21 system (NEPA GENE, Ichikawa-City, Chiba, Japan) in MG-63 and MG-63/DOX cells. Briefly, 1 × 10^6^ U-2 OS or U-2 OS/DOX cells were seeded in each well of six-well plates. On the next day, 75 pmol siRNA oligos mixed with 7.5 μL of Lipofectamine 2000 reagent and 485 μL of Opti-MEM (Life Technologies) were transfected into cells. Media were replaced after 4 hr. In MG-63 or MG-63/DOX cells, the Super Electroporator NEPA21 system was used. One million cells suspended in 90 μL Opti-MEM were transfected using 10 μL Opti-MEM containing 150 pmol siRNA oligos. After transfection, cells were transferred into six-well plates and allowed to adhere.

To develop DOX-resistant OS cell lines, 0.5 × 10^5^ cells were plated per well in a 12-well plate. Twelve hours later, cells were pretreated with 1 mL of complete media in the presence of 5 μg/mL Polybrene (sc-134220, Santa Cruz Biotechnology) for 5-min. Subsequently, cells were treated with shRNA lentiviral particles for GRP78 (15 μL/well containing 7.5 × 10^4^ infectious units of virus (IFU)) and control (15 μL/well containing 7.5 × 10^4^ IFU). Fresh media without Polybrene were placed on each infected well after 24 hr.

For Knockdown of GRP78 in tumor xenograft experiments, MG-63 cells were infected with the lentiviral constructs as described above. The stable clones were selected as previously described [42]. ShRNA knockdown efficiency of *in vitro* and *in vivo* experiments was shown in Figure 5A and 5E.

### Tumor xenografts

All animal experiments were approved by the Institutional Animal Care and Use Committee of China Pharmaceutical University. Four-week old nude female mice (BALB/c, nu/nu) were purchased from the Slac Laboratory Animal of Chinese Academy of Science (Shanghai, China). Three million MG-63 cells were stably transfected with shGRP78, or control lentiviral particles were suspended in 1:2 PBS/Matrigel (BD Biosciences, San Diego, CA, USA) in a total volume of 200 μL and subcutaneously injected into the right forelimb of each mouse. When the mean tumor volume reached approximately 100 mm^3^, 30 mice were randomized into five groups (*n* = 6/group) with approximately equivalent tumor volumes between groups. In accordance with the *in vitro* experiment, the minimum effective dose of MK2206 (60 mg/kg) was used [20]. In brief, MK2206 was formulated in 30% Captisol (Cydex, La Jolla, CA, USA) and administered by oral gavage thrice a week. DOX (hydrochloride) was administered by intraperitoneal injection with the dose of 2 mg/kg once every 4 days. The control group was treated with 30% Captisol by oral gavage thrice a week. Tumor growth was measured with calipers every other day, and tumor volume was calculated using the following formula: volume (mm^3^) = length × width^2^ × 0.5. After 21 days, the animals were anesthetized with 1.5% isofluorane-air mixture and killed by cervical dislocation. Xenografts were then removed and weighed.

### IHC

IHC staining analysis was performed as previously described [43, 44]. The slides were incubated overnight at 4°C with antibodies at a dilution of 1:800 for MDR1/ABCB1 (P-gp), 1:200 for BiP (GRP78), and 1:50 for Phospho-Akt (Ser 473) (all from Cell Signaling Technology). The following staining procedure was performed at 37°C for 60 min with secondary streptavidin-horseradish peroxidase-conjugated antibody (Santa Cruz Biotechnology). Image-Pro Plus 6.0 Software (Media Cybernetics, Bethesda, MD, USA) was used to analyze the expression of proteins.

### Development of DOX-resistant OS cell lines

To evaluate the effect of GRP78 inhibition and MK2206 on preventing the development of DOX resistance, we seeded U-2 OS or MG-63 cells into 12-well culture plates at 0.5 × 10^5^ cells per well overnight. Three parallel wells in each group were treated simultaneously. Subsequently, cells were transfected with the lentiviral constructs as described above. Fresh media were placed on each infected well after 24 hr, and cells were treated with DOX and/or 30 nM MK2206. The initial concentration of DOX was 5 nM. When cell growth reached 90% confluence, cells were harvested and reseeded at a density of 0.5 × 10^5^ cells/well, and the DOX concentration increased [45].

### Statistics

All quantitative results were expressed as mean ± SD of the data from at least three experiments conducted in a parallel manner. Statistical analysis was performed using GraphPad Prism 5 software.

## SUPPLEMENTARY MATERIALS FIGURE


