# Supplementary material for: Combining GRP78 suppression and MK2206-induced Akt inhibition decreases doxorubicin-induced P-glycoprotein expression and mitigates chemoresistance in human osteosarcoma
Source: Oncotarget. 2016 Jul 28;7(35):56371–82. doi: 10.18632/oncotarget.10890 (PMC5302920; doi:10.18632/oncotarget.10890)
Supplement: Supplementary file 1 [file oncotarget-07-56371-s001.pdf]

Combining GRP78 suppression and MK2206-induced Akt inhibition decreases doxorubicin-induced P-glycoprotein expression and mitigates chemoresistance in human osteosarcoma

Supplementary Materials

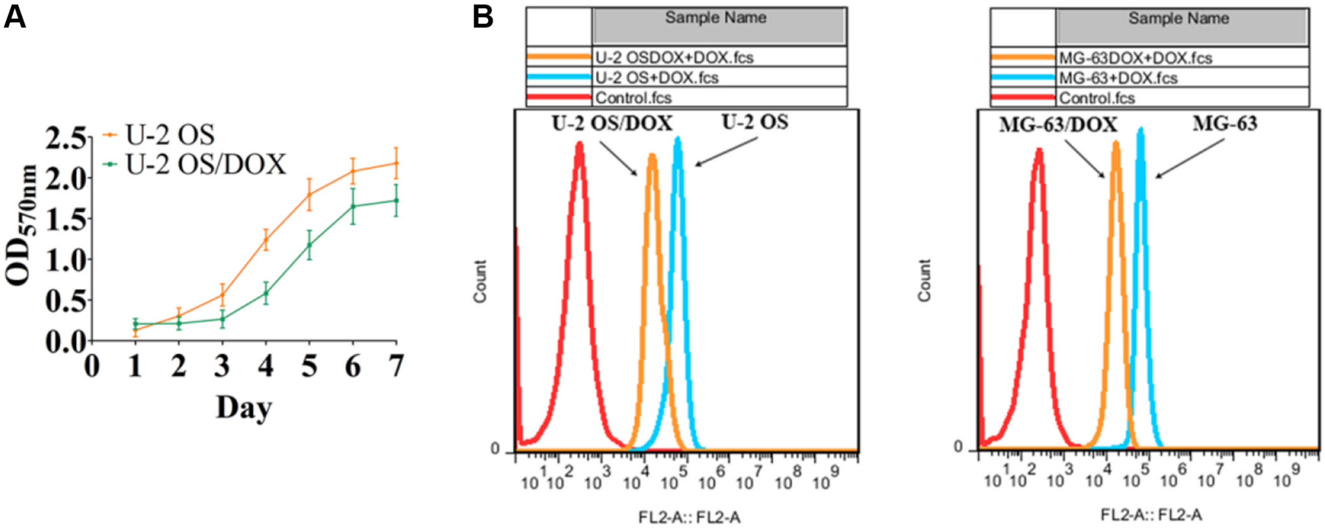

Supplementary Figure S1: (**A**) U-2 OS or U-S OS/DOX cells were seeded into 96-well culture plates and incubated for 1 to 7 days respectively. MTT assay was performed to measure the cell growth curve ($n = 3$). Data are represented as mean ± SD. (**B**) DOX accumulation assay. OS parental sensitive or resistant cells were incubated for 3 h in a medium containing 10 µM DOX, washed twice with PBS and suspended in PBS for Fluorescence activated Cell Sorting. OS parental cells without DOX incubation was used as the negative control.
